# Supplementary material for: The microbiome biomarkers of pregnant women’s vaginal area predict preterm prelabor rupture in Western China
Source: Front Cell Infect Microbiol. 2024 Oct 31;14:1471027. doi: 10.3389/fcimb.2024.1471027 (PMC11560878; doi:10.3389/fcimb.2024.1471027)
Supplement: Supplementary file 1 [file DataSheet1.zip › compare_1/Community/Pieplot/family/sample/P18.family.pieplot.pdf]

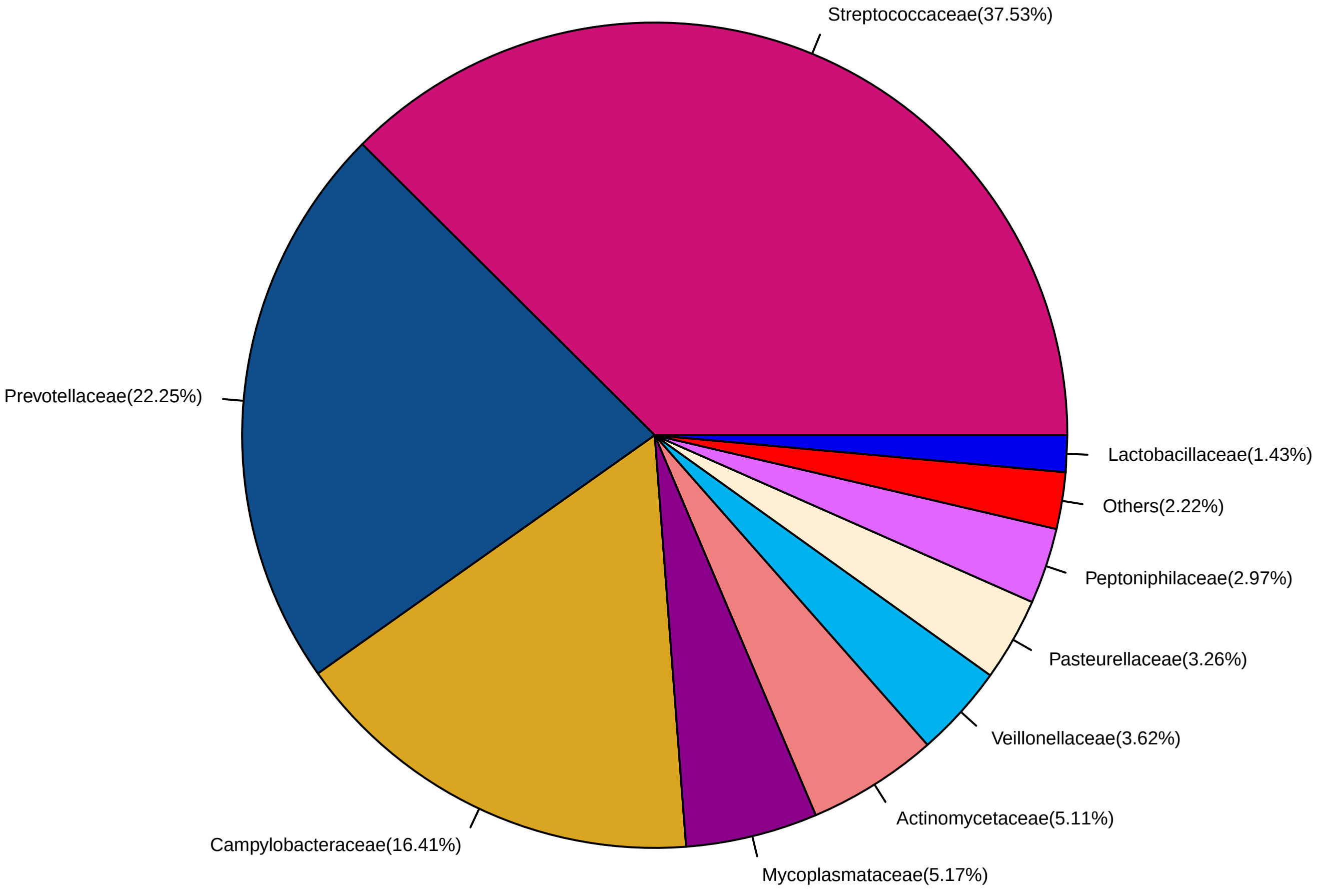
Streptococcaceae(37.53%)
Prevotellaceae(22.25%)
Lactobacillaceae(1.43%)
Others(2.22%)
Peptoniphilaceae(2.97%)
Pasteurellaceae(3.26%)
Veillonellaceae(3.62%)
Actinomycetaceae(5.11%)
Campylobacteraceae(16.41%)
Mycoplasmataceae(5.17%)
